# An Improved Detection of Circulating Tumor DNA in Extracellular Vesicles-Depleted Plasma

**DOI:** 10.3389/fonc.2021.691798

**Published:** 2021-06-11

**Authors:** Li Sun, Meijun Du, Manish Kohli, Chiang-Ching Huang, Xiaoxiang Chen, Mu Xu, Hongbing Shen, Shukui Wang, Liang Wang

**Affiliations:** ^1^ Laboratory Medicine Center, the Second Affiliated Hospital, Nanjing Medical University, Nanjing, China; ^2^ Department of General Clinical Research Center, Nanjing First Hospital, Nanjing Medical University, Nanjing, China; ^3^ Department of Pathology, Medical College of Wisconsin, Milwaukee, WI, United States; ^4^ Department of Epidemiology and Biostatistics, Jiangsu Key Laboratory of Cancer Biomarkers, Prevention and Treatment, Collaborative Innovation Center for Cancer Medicine, School of Public Health, Nanjing Medical University, Nanjing, China; ^5^ Division of Oncology, University of Utah Huntsman Cancer Institute, Salt Lake City, UT, United States; ^6^ Zilber School of Public Health, University of Wisconsin, Milwaukee, WI, United States; ^7^ Department of Tumor Biology, H. Lee Moffitt Cancer Center, Tampa, FL, United States

**Keywords:** ctDNA, liquid biopsy, plasma, exosome, copy number variation

## Abstract

Circulating tumor DNA (ctDNA) in plasma has been used as a biomarker for cancer detection and outcome prediction. In this study, we collected the five precipitates (fractions 1–5) and leftover supernatant plasma component (fraction 6) by a sequential centrifugation in plasma samples from nine small cell lung cancer (SCLC) patients. The fractions 3, 5 and 6 were large vesicles, exosomes and extracellular vesicles (EVs)-depleted plasma, respectively. Fragment size analysis using DNAs from these fractions showed dramatical differences from a peak of 7–10 kb in fraction 1 to 140–160 bp in fraction 6. To determine ctDNA content, we performed whole genome sequencing and applied copy number-based algorithm to calculate ctDNA percentage. This analysis showed the highest ctDNA content in EV-depleted plasma (average = 27.22%), followed by exosomes (average = 22.09%) and large vesicles (average = 19.70%). Comparatively, whole plasma, which has been used in most ctDNA studies, showed an average of 23.84% ctDNA content in the same group of patients. To further demonstrate higher ctDNA content in fraction 6, we performed mutational analysis in the plasma samples from 22 non-small cell lung cancer (NSCLC) patients with known EGFR mutations. This analysis confirmed higher mutation detection rates in fraction 6 (14/22) than whole plasma (10/22). This study provides a new insight into potential application of using fractionated plasma for an improved ctDNA detection.

## Introduction

Cancer is a serious public burden with an estimation of 1,898,160 new cancer cases and 608,570 cancer deaths in the United States in 2021 (1). To reduce cancer-related morbidity and mortality, more effective approaches in diagnosis and treatment are urgently needed. It is well known that genomic abnormalities are not only hallmarks of cancers but also in evolution during cancer progression (2). Due to intratumor heterogeneity, however, genomic sequencing from a single tumor biopsy may not fully capture the genomic profile of tumors (3). Moreover, tissue biopsy is limited on tissue availability and sampling frequency. It may increase patients’ risk of complication because of the invasive procedure. To address these issues, analysis of circulating cell-free DNA (cfDNA) in blood has been used as a non-invasive method for molecular characterization of tumor genome variations. This blood-based approach has been referred to as liquid biopsy and has demonstrated great potential in cancer diagnosis and outcome prediction (4–6).

Due to lack of adequate oxygen and nutrition, rapidly growing tumor cells often become stressed, and experience apoptosis and necrosis. DNA fragments released from these dead cells eventually end up in circulating blood (7). In patients with cancer, a fraction of cfDNA is tumor-derived and is termed circulating tumor DNA (ctDNA). Analysis of ctDNA has an advantage of identifying genomic alterations that are specific to tumor (8, 9). Interestingly, ctDNA has also been reported in isolated extracellular vesicles (EVs) (10–12). Analysis of vesicles-associated nucleic acids for BRAF, KRAS, and EGFR mutations has shown higher sensitivity compared to plasma ctDNA in non-small cell lung cancer (NSCLC) patients (13). Microvesicles isolated from plasma of NSCLC patients can be used for EGFR genotyping for the detection of drug-resistance mutations, demonstrating improved concordance with tumor tissue compared to a conventional ctDNA (14). Additionally, exosomes from patients with metastatic pancreatic cancer showed a higher mutant *KRAS* allele frequency than exosomes from patients with local disease (15). These studies suggest that EVs may enrich ctDNA and may be used as a preferred source of material for cancer biomarker discovery. However, a recent study showed that the extracellular DNA may not be associated with exosomes, but could instead be co-purified with the small EV fraction during standard isolation protocols (16). Nevertheless, these studies suggest that EVs and/or their co-precipitates enrich ctDNA and may be used to increase sensitivity of cancer biomarker detection.

To systematically determine DNA size distribution and ctDNA content in different fractions of plasma, in this study, we collected plasma from nine small cell lung cancer (SCLC) patients with known high ctDNA content (17). We performed five consecutive centrifugations, collected each of precipitates and analyzed DNA size distribution in each collection. We also performed low-pass whole genome sequencing and estimated ctDNA content using a novel copy number-based algorithm in each of these fractions. In a separate set of plasma samples consisting of 22 non-small cell lung cancer (NSCLC) patients with known EGFR mutations, we compared the mutation detection rate in the fractionated plasma and whole plasma.

## Materials and Methods

### Patients and Plasma Collection

We selected nine SCLC patients whose plasma demonstrated relatively high ctDNA content based on our previous study (17), and 22 NSCLC patients with known EGFR mutations in tumor tissues. We collected the plasma samples from the Medical College of Wisconsin Tissue Bank and the Second Affiliated Hospital of Nanjing Medical University. Original plasma samples (platelet-rich) were prepared by one time 3,000 rpm for 10 min as previously described (17–19). All samples were uniformly processed and stored at −80°C prior to this study. Cancer diagnosis was confirmed in all cases by routine histopathologic examination. All participants provided written informed consent. This study was approved by the Medical College of Wisconsin Institutional Review Broad and the Research and Ethical Committee of the Second Affiliated Hospital of Nanjing Medical University.

### Characterization of Fractions 3 (Large EVs) and 5 (Exosomes)

**Figure 1:**
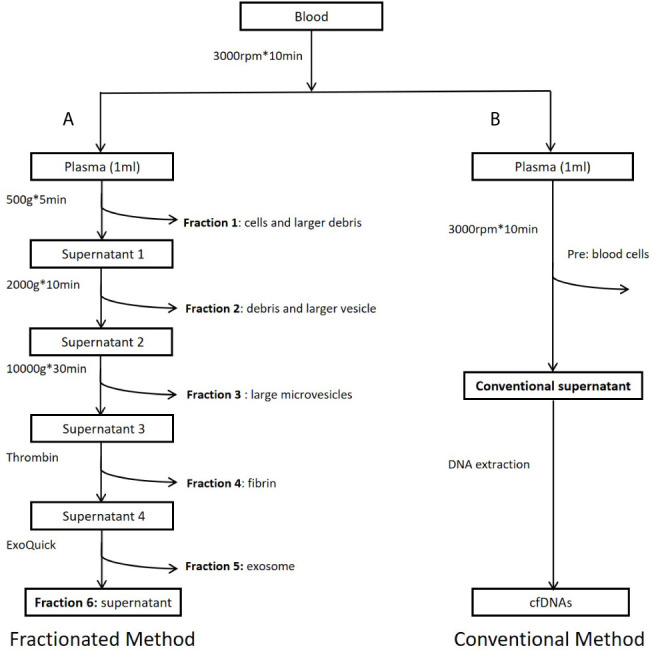
Workflow of study design. **(A)** Preparation of plasma fractions by five consecutive centrifugations. **(B)** Conventional plasma cfDNA extraction. Fraction 1: Precipitates after centrifugation at 500g. Major components of this fraction are some cells and large cell debris. Fraction 2: Precipitates after centrifugation at 2,000*g* from fraction 1 supernatant. Major components of this fraction are small cell debris and large vesicles, such as apoptotic bodies. Fraction 3: Precipitates after centrifugation at 10,000*g* from fraction 2 supernatant. Major component of this fraction is large microvesicles. Fraction 4: Precipitates after centrifugation at 10,000*g* from fraction 3 supernatant treated with Thrombin at room temperature for 5 min (9.5 ul Thrombin per 950 ul sample). Major component of this fraction is fibrin. Fraction 5: Precipitates after centrifugation at 1,500*g* from fraction 4 supernatant treated with Exoquick (System Biosciences, Mountain View, CA, USA) at 4°C overnight. Major component of this fraction is believed to be exosomes. Fraction 6: Supernatant from fraction 5. This fraction is the leftover supernatant after removing precipitates from five consecutive centrifugations.

A total of 10 ml pooled plasma from 20 healthy individuals (0.5 ml of each) was used for fractions 3 and 5 preparation (**Figure 1** for detail). Transmission electron microscopy (TEM), Nanosight and flow cytometry were used to characterize the two fractions. The fractionated samples were first fixed in 2.5% glutaraldehyde solution for 2 h. About 10 μl of the diluted mixtures were then transferred to a cleaned copper net and images were obtained by TEM (JEM-1010, JEOL, Japan) after staining with 2% phosphotungstic acid solution. For Nanosight analysis, fractionated samples were diluted 2,000-fold in PBS for size distribution analysis using a Zetasizer Nano ZS (Malvern Instruments Ltd, UK). For flow cytometry analysis, the fractions 3 and 5 were first resuspended in 100 μl PBS and then incubated with anti-CD63 and anti-CD81 specific monoclonal antibodies (BD Biosciences, San Jose, CA, USA) with fluorescent direct labeling. BD Accuri C6 Flow Cytomenter (BD Accuri, San Jose, CA, USA) was used to examine the characteristic protein markers of the two fractions.

### DNA Extraction and Quantification

For each SCLC patient, 1 ml platelet-rich plasma (one spin at 3,000 rpm for 10 min) was used for fraction separation. For each NSCLC patient, 2 ml platelet-poor plasma (double spins at 3,000 rpm for 10 min) was used for DNA extraction and subsequent mutational analysis. DNAs from all samples were extracted using DNA Blood Mini Kit (Qiagen, Valencia, CA, USA). Final DNA eluent (50 µl) was quantified by Qubit 2.0 Fluorometer (Life Technologies, Carlsbad, CA, USA). High Sensitivity DNA Analysis Chip (Agilent Technologies, Santa Clara, CA, USA) was used to examine the DNA size distribution of different fractions. The extracted DNA was stored at −20°C until use.

### Library Preparation and Whole Genome Sequencing

DNA libraries were prepared using a ThruPLEX DNA-seq Library Kit (Takara Bio, Mountain View, CA) according to the manufacturer’s instructions. About 0.5–1 ng DNA was used for library preparation including end repair, adaptor addition, and 15 cycles of high-fidelity amplification. Following amplification, libraries were purified using a 1:1 ratio of DNA sample to Agencourt AMPure XP Beads (Beckman Coulter, Indianapolis, IN, USA). The library quality and insert size were examined using High Sensitivity DNA Analysis Chip. Sequencing Libraries were diluted to a concentration of 10 nM and every 12 index libraries were pooled for 50 bp single-read sequencing on a HiSeq2500 Sequencing System (Illumina, San Diego, CA, USA).

### Copy Number Variation (CNV) and ctDNA Content Calculation

Raw sequencing data (fastq files) were first mapped to the human reference genome (NCBI37/hg19) using SeqMan NGen 12 (DNASTAR, Madison, WI, USA) and assembled in Partek Genomics Suite (St. Louis, MO, USA). The mapped reads were then binned into 1 Mb genomic bins and rescaled to 10 million reads after excluding sex chromosomes. Read count in each genomic window was normalized to mean read count from 33 healthy controls as previously described (19). The resulting ratios were further transformed with log2 and adjusted for GC content (20). The fully normalized log2 ratios in genomic bins were subjected to segmentation using the copy number analysis method (CNAM) algorithm (Golden Helix, Bozeman, MT, USA). To estimate ctDNA content, we developed a CNV-based algorithm to quantify ctDNA percentage in plasma cfDNA (21). In these studies, we used mean log2 values of genomic segments generated from CNAM algorithm for ctDNA content calculation. Segment sizes were evaluated to test ctDNA content stability. We selected mean log2 values from most significant deletion segments (>20 Mb in size) in each patient and calculated ctDNA content by 1–2^segment log2 ratio^ (21).

### EGFR Mutational Analysis

For 22 NSCLC patients with known EGFRE19del/L858R mutations in tumor tissues, we applied ARMS-PCR method and tested their mutational status in fraction 6 and platelet-poor plasma samples using EGFR Mutations Detection Kit (Amoy Diagnostics, Xiamen, Fujian, China). Mutational analysis was performed on the ABI 7500 Real-Time PCR System (Thermo Fisher Scientific, Foster City, CA, USA). PCR was prepared by mixing 5 µl DNA with 5 µl control reaction mix or mutation mix (E19del, L858R). PCR was set up as follows: 95°C/5 min; 15 cycles of 95°C/25 s, 64°C/20 s and 72°C/20 s; 31 cycles of 93°C/25 s, 60°C/35 s and 72°C/20 s. The signal is collected at 60°C in the third stage.

## Results

### Plasma Fractions

To estimate cfDNA size and ctDNA content from different plasma fractions, we selected nine SCLC patients (**Table S1**) who have been previously analyzed and showed relatively high tumor burden (17). As described in previous publications, increasing centrifugation speed may generate sequential precipitations of different components including cells, cell debris, larger vesicles, apoptotic bodies, and microvesicles (11, 12, 22, 23). Based on these publications, we performed sequential centrifugation using 1 ml plasma sample/patient and collected six fractions for separate cfDNA extraction. **Figure 1** shows overall workflow of this study. To test higher ctDNA content in a plasma fraction, we compared mutation detection rate between the plasma fraction and whole plasma in 22 NSCLC patients with known EGFR mutations in tumor tissues.

### Characterization of Extracellular Vesicles in Fractions 3 and 5

**Figure 2:**
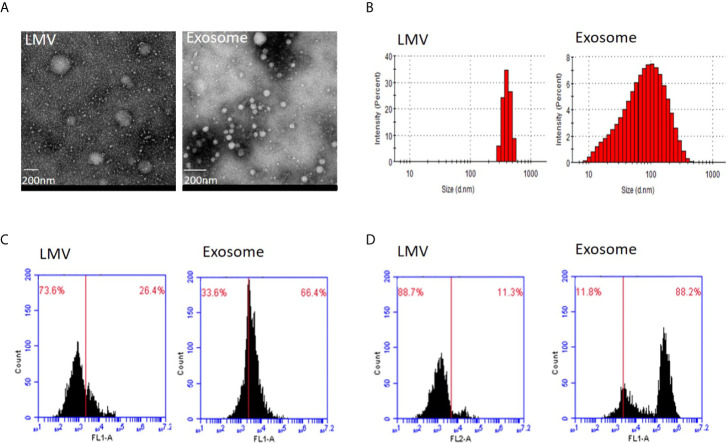
Identity analysis of fraction 3 (large microvesicles) and fraction 5 (exosomes). **(A)** Transmission electron microscopy. The round shape of large microvesicles (LMV) and exosomes by negatively staining the background with phosphotungstic acid. The bar represents 200 nm. **(B)** Nanosight analysis. Particle sizes of fractions 3 and 5 are different with 405.5 nm and 100.3 nm in the main peak value, respectively. **(C, D)** Flow cytometry of characteristic protein analysis. Results of CD63 **(C)** and CD81 **(D)** positive ratio show 26.4 and 11.3% in fraction 3, and 66.4 and 88.2% in fraction 5, respectively.

We first applied transmission electron microscopy and nanoparticle tracking analysis (Nanosight) to estimate vesicle size in the fractions 3 and 5 (**Figures 2A, B**). This analysis showed that all particles in fraction 3 were within the range 200–600 nm with peak size at 405.5 nm while 83.7% particles in fraction 5 were within 20–200 nm with main size at 100.3 nm. We then applied flow cytometry to examine characteristic protein markers in the two fractions. This analysis showed that CD63 and CD81 positive ratio were 26.4 and 11.3% in fraction 3, and 66.4 and 88.2% in fraction 5, respectively (**Figures 2C, D**). Clearly, the fraction 3 is featured as large microvesicles while fraction 5 is featured as exosomes.

### DNA Yield in Each Plasma Fraction

**Figure 3:**
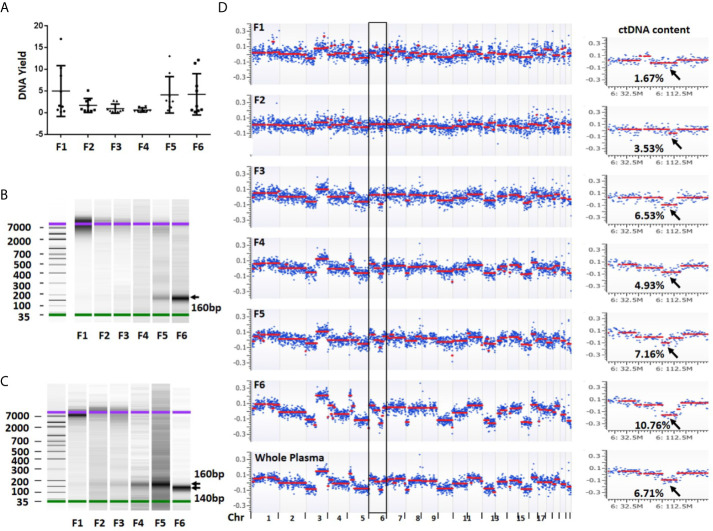
DNA yield, size and overall view of genomic alterations in six fractions collected from a sequential centrifugation of 1 ml plasma sample. **(A)** DNA yield (ng) from six different fractions. **(B)** DNA size (bp) distribution in six plasma fractions of patient 1. **(C)** DNA size (bp) distribution in six plasma fractions of patient 7. **(D)** Overall view of genomic alterations in six plasma fractions and their corresponding whole plasma from patient 1. Segmentation-based copy number variation analysis shows different genomic variations across chromosomes 1–22. Most significant segments losses (arrows) on chromosome 6 were used to calculate ctDNA content. The log2 ratio scale in y axis was from −0.4 to 0.4. F1–6 represent fractions 1-6, respectively.

To evaluate DNA yield from each plasma fraction, we isolated DNA and quantified the DNA concentration using a high sensitive Qubit assay in a total of 54 fractionated biospecimens. From 1 ml starting plasma, an average yield of each individual fractions was 5.03 ng (median = 1.66 ng, range 0.23–17.01 ng) in fraction 1, 1.73 ng (median = 0.86 ng, range 0.47–5.15 ng) in fraction 2, 0.99 ng (median = 0.50 ng, range 0.18–2.84 ng) in fraction 3, 0.68 ng (median = 0.40ng, range 0.18–1.48 ng) in fraction 4, 4.17 ng (median = 2.65 ng, range 0.58–13.05 ng) in fraction 5 and 4.28 ng (median = 1.55 ng, range 0.22–12.15 ng) in fraction 6. Although the average DNA yields in fractions 1, 5 and 6 were among top three and accounted for 79.9% of all DNA yields, their variations were also among the top three. In contrast, fractions 2–4 showed relatively low but stable DNA yield (**Figure 3A**).

### DNA Size Distribution in Different Plasma Fractions

To investigate DNA size distribution, we measured each of the six DNA samples using Agilent Bioanalyzer. This analysis revealed distinct peak sizes in different fractions. The fraction 1 showed a peak size of 7,000–10,000 bp which was gradually reduced in fractions 2–3. Although barely seen in fraction 1, the density of a smaller fragment at ~160 bp was gradually intensified from fractions 3 to 6 (**Figure 3B**). In some samples, the fraction 6 showed a peak size at ~140 bp (**Figure 3C**). The fragment sizes are similar to the DNA length of a mono-nucleosome (∼147 bp) (23). Overall, we observed clear trend that the larger fragment (~10,000 bp) was slowly diminished from fractions 1 to 3 while smaller fragment (~160 bp) was gradually increased from fractions 4 to 6. Clearly, larger DNA fragments in fractions 1 and 2 are more likely derived from genomic DNA contamination of cell debris and platelets.

### ctDNA Content in Different Fractions of Plasma

To estimate ctDNA content (defined as ctDNA percentage in a total cfDNA) from each plasma fraction, we first performed low-pass whole genome sequencing and received approximately 20 million (range 9.4–42.1 million) mappable reads per fraction (**Table S2**). We then performed log2 ratio-based segmentation analysis using 1 Mb genomic windows. This analysis showed a significant change of detectable copy number among different fractions. In general, the fractions 1 and 2 might show detectable copy number variations (CNVs) but call confidence was relatively low. In contrast, the fractions 3 (large EVs), 5 (exosomes) and 6 (EVs-depleted plasma) were more likely to demonstrate detectable CNVs with high confidence call (**Figure 3D**).

**Figure 4:**
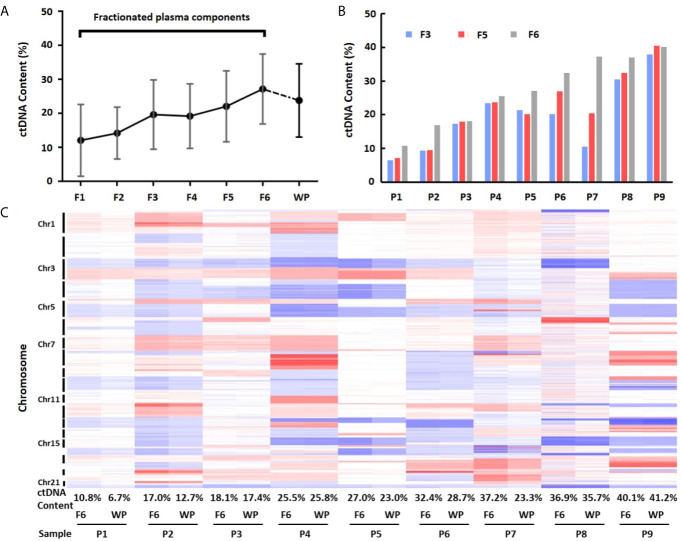
Differences of ctDNA content in fractionated plasma components and whole plasma. **(A)** Overall view of ctDNA content from fractionated plasma components and whole plasma. The average ctDNA content is the highest in EVs-depleted fraction 6. **(B)** Differences of ctDNA content among fractions 3, 5 and 6. **(C)** Heatmap of log2 ratio in 1 Mb genomic window across chromosomes 1–22 in fraction 6 (F6) and whole plasma (WP) from nine patients (P1–P9). Red color represents copy number gain, while blue represents loss. Intensity of the color is proportional to the value of log2 ratio and reflects the weight of ctDNA in overall background cfDNA.

To calculate ctDNA content, we selected mean value from most significantly deleted segments in each patient and estimated ctDNA proportion in each individual fraction (21). This analysis showed that the average ctDNA content was 12.12% (median = 9.30%, range 0.39–27.09%) in fraction 1, 14.25% (median = 16.93%, range 3.53–22.82%) in fraction 2, 19.70% (median = 20.18%, range 6.53–37.94%) in fraction 3, 19.23% (median = 18.42%, range 4.93–38.60%) in fraction 4, 22.09% (median = 20.45%, range 7.16–40.51%) in fraction 5, and 27.22% (median = 27.04%, range 10.76–40.12%) in fraction 6. Clearly, ctDNA content in EVs-depleted fraction 6 was the highest among the six components. Fractions 5 (exosome) and 3 (large EVs) showed the 2nd and 3rd highest ctDNA content, respectively (**Figures 4A, B** and **Table S3**). We also compared the fractionated plasma DNA to platelet-poor plasma DNA for their ctDNA content differences. The ctDNA content in platelet-poor plasma showed an average of 23.84% (median = 23.34%, range 6.71–41.22%), further support that the fraction 6 has the highest ctDNA content among all plasma fractions and whole platelet-poor plasma.

To further demonstrate ctDNA content difference between fraction 6 and whole plasma, we performed clustering analysis using GC-corrected log2 ratio as input. Although fraction 6 and platelet-poor plasma from the same patients clustered perfectly across all chromosome regions, the heatmap showed clear intensity differences in most regions showing CNVs (**Figure 4C**). Of nine cases, seven showed higher intensity (hence, higher ctDNA content) in fraction 6 than in whole plasma. For example, based on mean absolute log2 ratios at these selected genomic segments (**Table S4**), we estimated that ctDNA content in patient 7 was 37.2% in fraction 6 while 23.3% in platelet-poor plasma, indicating 13.9% more ctDNA content in fraction 6 than whole plasma sample in the patient.

### Detection of EGFR Mutations in Fractionated Plasma and Platelet-Poor Plasma

Since fraction 6 showed the highest ctDNA content, we hypothesized that the fraction 6 had higher sensitivity in mutation detection. To test this, we selected 22 non-small cell lung cancer (NSCLC) patients with known EGFR E19del/L858R mutations in tumor tissues (**Table S5**). The positive percentage of serum tumor biomarkers including CEA (Carcino-embryonic antigen) and CYFRA 21-1(Cytokeratin-19-fragment) was 36.4 and 40.9%, respectively, in these NSCLC patients. We applied the amplification-refractory mutation system (ARMS)-PCR assays to detect these mutations in the DNAs derived from the fraction 6 and platelet-poor plasma. Among the 22 patients, we identified EGFR mutations that matched to tumor tissues in 14 of fraction 6 samples and 10 of platelet-poor plasma samples. Sensitivity of the EGFR mutation detection was 63.6% (95% CI: 40.8 to 82.0%) in fraction 6 and 45.5% (95% CI: 25.1 to 67.3%) in platelet-poor plasma, respectively (**Table 1**). This result suggests that compared to traditionally used platelet-poor plasma, the fraction 6 derived from a series of centrifugations including removal of EVs may improve EGFR mutation detection.

**Table 1:** Comparison of the EGFR mutation status between fraction 6 DNA and cell free DNA in NSCLC patients.

EGFR genotype	Tissue	Plasma (n = 22)			
		Fraction 6 DNA		cfDNA	
		Mutant type	Wild type	Mutant type	Wild type
**Mutant type**	22 (100.0%)	14 (63.6%)	0	10 (45.5%)	0
**Wild type**	0	0	8 (36.4%)	0	12 (54.5%)
**Sensitivity (%) (95% CI)**		63.6% (40.8–82.0)		45.5% (25.1–67.3)	
**Specificity (%) (95% CI)**		NA		NA	

CI, Confidence Interval; NA, not available.

## Discussion

It is well known that ctDNAs are detectable in plasma samples of peripheral blood (24–26). The ctDNAs appear to demonstrate unique DNA fragmentation pattern and smaller fragment size (27). However, effect of plasma preparation methods on ctDNA content has not been reported. In this study, we isolated DNA from six fractions of plasma samples by multiple physical and chemical precipitations. We applied low-pass whole genome sequencing technology to determine CNVs for ctDNA content estimation. Our results showed that DNA fragment size and ctDNA content varied among the six fractions with fraction 6 showing enrichment of smaller DNA fragments and tumor-derived cfDNA. Fraction 6 also showed higher sensitivity in mutation detection than whole (unfractionated) plasma. These results suggest that plasma preparation before DNA extraction is an important step for sensitive detection of low level ctDNA in peripheral blood.

By separating whole plasma into six fractions, we were able to compare DNA yield, size distribution and ctDNA content differences among these fractionated samples. For fractions 1–3, ~10,000 bp DNA fragments are dominant but total DNA yields are gradually decreased. Since fractions 1–2 are primarily composed of contaminated cell debris, platelets and larger vesicles such as apoptotic bodies, it is not surprised to see higher molecule weight DNA fragments. The fraction 3 is believed to contain primarily large EVs, which have shown predominantly large size (~10,000 bp) dsDNA by chip-based capillary electrophoresis (11), which is consistent with our observation. Additionally, a recent report showed that centrifugation protocols had an effect on DNA integrity (28). This study reported longer DNA fragments almost exclusively in CPBasic Fraction (plasma after 400*g* for 10 min centrifugation) and CPAdBasic_P Fraction (pellet after 400*g* for 10 min and max speed for 1 min), which is also consistent with our observation. Fractions 4–5 are dominated by 160 bp fragments. This DNA size is similar to commonly reported cfDNA and is corresponding to the size of chromatosomes (nucleosome + linker histone; ∼167 bp) (23). Fraction 4 is thrombin-precipitated fibrin. Thrombin acts as a serine protease that converts soluble fibrinogen into insoluble strands of fibrin, as well as catalyzing many other coagulation-related reactions. Fraction 5 is derived from Exoquick-precipitated exosomes and other small EVs. Studies have shown that the exosomes contain DNA from parent tumor cells (10–12). Interestingly, DNA sizes from fraction 6 seem sample-dependent with some samples being at ~160 bp while others showing ~140 bp. Fraction 6 is the leftover supernatant after five consecutive precipitations and can be considered as EVs-depleted plasma. Clearly, the smaller mono-nucleosome derived DNA has been preserved after multiple centrifugations. Further study is needed to determine whether the mono-nucleosome sized DNA is free in true free state or in histone-bound state.

By comparing different plasma fractions, we observed a clear trend of higher ctDNA content in fractions 3 (large EVs), 5 (exosomes) and 6 (EVs-depleted plasma). Consistent with our findings, a previous study showed that large vesicles from cancer patients enriched ctDNA (11). Another study showed that DNA from nanoscale vesicles (30–220 nm, the size of exosomes) is better than whole plasma cfDNA for mutation detection in early stage NSCLC (12). It is known that ctDNA tends to be shorter than normal cfDNA in plasma (29). An animal model-based study demonstrated that the most common fragment length of ctDNA was 134–144 bp, which is significantly smaller than the most common 167 bp fragment present in noncancer cfDNA (30). Since fraction 6 has smaller fragments (∼140 bp) than any other fractions, it may be one reason to explain why fraction 6 shows an increased ctDNA content and thus higher sensitivity in mutation detection. Additionally, detection of EGFR mutations in plasma samples of NSCLC patients are predictive of survival and resistance to EGFR TKI (31). Therefore, our results strongly support that the enriched ctDNA in fraction 6 will increase mutation detection sensitivity and facilitate identification of tumor-specific biomarkers.

An innovative feature of this study is the assessment of ctDNA content in multiple fractions of plasma. Since all fractions were derived from the same 1 ml of plasma by consecutive centrifugations, we were able to directly compare ctDNA contents in different fractions from the same patients. Another feature is ctDNA content estimation using a novel algorithm. To determine ctDNA content, mutant allele frequency is commonly used. However, it is difficult to calculate ctDNA content when cfDNA input is low. In this study, we applied a CNV-based algorithm to estimate ctDNA content (21). The CNV-based method uses an average log2 ratio values across multiple genomic bins (windows). Therefore, the estimate is expected to be more stable when compared to single mutant allele-based method. Additionally, we selected patients with high tumor burden from our previous study (17). The high tumor burden is necessary to accurately determine ctDNA content and to demonstrate difference of the ctDNA content among these fractions. It is worth mentioning that the plasma fractionation may allow maximum use of valuable plasma samples for a wide variety of studies. For example, supernatant fraction of a plasma sample may be used for ctDNA-based genetic analysis while exosome fraction of the same plasma sample may be used for microRNA-based biomarker study. This approach resembles blood transfusion of components in clinic to efficiently use different blood fractions.

Although we observed significant ctDNA content differences in fractionated plasma components and showed higher sensitivity in mutation detection in EV-depleted plasma fraction, this study also has some limitations. First, we were not able to remove any possible DNA that may be co-purified with exosomes in fraction 5. A new study has shown that extracellular DNA could be co-purified with the small EV fraction during standard isolation protocols (16). Therefore, the origin of ctDNAs detected in fraction 5 needs further investigation. Second, we made double stranded DNA library for sequencing analysis. It seems that double stranded DNA might not be associated with exosomes or with any small EVs at all (32). Single strand DNA library preparation method may be needed to evaluate the presence of ctDNA in exosomes. Third, we tested plasma EGFR mutations in 22 patients only. Although the EV-depleted plasma fraction showed higher mutation detection rate than whole plasma (63.6% vs 45.5%), the difference did not reach statistical significance. Further study in large sample size is needed. Fourth, the current study used a sequential centrifugation process, which was involved in multiple pipetting and sample transfer, significantly increasing risk of sample contamination. Future study to optimize the centrifugation steps will help simplify the sample processing. Finally, because tumor genome is evolving during disease progression, it is interesting to analyze ctDNA content changes in different plasma fractions at different blood draw time points. Nevertheless, our study provided a new insight into potential application of fractionated plasma for an improved ctDNA detection. The result supports that different plasma fractions may enrich different types of tumor-associated molecules. Further understanding of DNA origins in different plasma fractions will facilitate cancer biomarker discovery.

## Conclusions

cfDNA from different fractions of plasma varies in fragmentation sizes and ctDNA contents. Due to its higher ctDNA content and increased sensitivity of mutation detection, the fraction 6 is the preferred source of material for ctDNA-based genomic analysis.

## Data Availability Statement

The datasets presented in this study can be found in online repositories. The names of the repository/repositories and accession number(s) can be found in the article/**Supplementary Material**.

## Ethics Statement

The studies involving human participants were reviewed and approved by the Medical College of Wisconsin Institutional Review Broad. The patients/participants provided their written informed consent to participate in this study. Written informed consent was obtained from the individual(s) for the publication of any potentially identifiable images or data included in this article.

## Author Contributions

Conceptualization, LS and LW. Methodology, LS and MD. Writing—original draft preparation, LS. Writing—review and editing. MK, LW, HS, SW, C-CH, XC, and MX. Funding acquisition, LW, SW, and LS. All authors contributed to the article and approved the submitted version.

## Funding

This research was funded by National Institute of Health (grant number R01CA212097) to LW, Key Project of Science and National Natural Science Foundation of China (No. 81972806) to SW, Key Project of Science and National Natural Science Foundation of China (No. 82002230), Medical Scientific Research Guidance Project of the Health Commission of Jiangsu (No. Z2020007), 789 Outstanding Talent Program of SAHNMU (No. 789ZYRC202080338), and International Exchange and Cooperation Foundation of Nanjing Medical University (No. C063, D009) to LS.

## Conflict of Interest

The authors declare that the research was conducted in the absence of any commercial or financial relationships that could be construed as a potential conflict of interest.
