# Supplementary material for: An Improved Detection of Circulating Tumor DNA in Extracellular Vesicles-Depleted Plasma
Source: Front Oncol. 2021 Jun 11;11:691798. doi: 10.3389/fonc.2021.691798 (PMC8226168; doi:10.3389/fonc.2021.691798)
Supplement: Supplementary file 1 [file DataSheet_1.docx]

Supplementary Material

Table S1. Clinical characteristics of SCLC patients

Table S2. Mappable reads per fraction of plasma in SCLC patients

Table S3. ctDNA content in six fractions and whole plasma from 9 SCLC patients

Table S4. Selected segments and absolute Log2 ratio from fraction 6 and whole plasma

Table S5: Clinical characteristics of NSCLC patients

Table S1. Clinical characteristics of SCLC patients

| Patient # | Sex | Ages | Stage | Chemo-  therapy | Radio-  therapy | Tobacco  (Pack years) |
| --- | --- | --- | --- | --- | --- | --- |
| 1 | F | 53 | Limited | Yes | No | 60 |
| 2 | M | 58 | Extensive | Yes | Yes | 50 |
| 3 | M | 58 | Extensive | Yes | Yes | 40 |
| 4 | M | 48 | Extensive | Yes | Yes | 30 |
| 5 | F | 69 | Extensive | Yes | Yes | 55 |
| 6 | F | 54 | Extensive | Yes | Yes | 35 |
| 7 | F | 54 | Limited | Yes | Yes | 33 |
| 8 | F | 45 | Limited | Yes | Yes | 13 |
| 9 | M | 55 | Extensive | Yes | Yes | 20 |

These patients had median age of 54 (range 45-69) including 5 females and 4 males. The clinical stage of these patients included 3 limited and 6 extensive. All patients experienced with chemotherapy and 8 of them also had radiation therapy. Median tobacco pack year was 35 (range 13-60).

Table S2. Mappable reads per fraction of plasma in SCLC patients

| Sample ID | Total reads | Mappable reads | Mappable ratio |
| --- | --- | --- | --- |
| P1-fraction1 | 20442969 | 17175283 | 0.840155997 |
| P1-fraction2 | 11474757 | 10077180 | 0.878204218 |
| P1-fraction3 | 14035063 | 11893832 | 0.847437023 |
| P1-fraction4 | 15539433 | 13773809 | 0.886377836 |
| P1-fraction5 | 31971570 | 28871336 | 0.903031537 |
| P1-fraction6 | 23580728 | 21372049 | 0.906335419 |
| P2-fraction1 | 25522864 | 20627072 | 0.808180148 |
| P2-fraction2 | 15195693 | 13257167 | 0.872429247 |
| P2-fraction3 | 20893544 | 16222064 | 0.776415145 |
| P2-fraction4 | 13388590 | 9627694 | 0.719096933 |
| P2-fraction5 | 18311831 | 16596380 | 0.906320073 |
| P2-fraction6 | 22384564 | 19966437 | 0.89197346 |
| P3-fraction1 | 26147808 | 23540830 | 0.900298411 |
| P3-fraction2 | 15242893 | 13702321 | 0.898931784 |
| P3-fraction3 | 15044349 | 13421764 | 0.892146546 |
| P3-fraction4 | 13469544 | 11990248 | 0.890174753 |
| P3-fraction5 | 38226963 | 34609220 | 0.905361485 |
| P3-fraction6 | 36679421 | 33155195 | 0.903918167 |
| P4-fraction1 | 39182087 | 35118290 | 0.896284315 |
| P4-fraction2 | 16103926 | 14304720 | 0.888275319 |
| P4-fraction3 | 13322074 | 10716079 | 0.804385188 |
| P4-fraction4 | 13196882 | 11760367 | 0.891147394 |
| P4-fraction5 | 40391129 | 36675350 | 0.908005072 |
| P4-fraction6 | 38608446 | 34869449 | 0.903155983 |
| P5-fraction1 | 36106277 | 32189506 | 0.89152105 |
| P5-fraction2 | 16731382 | 14682459 | 0.877540122 |
| P5-fraction3 | 13993081 | 12343430 | 0.882109523 |
| P5-fraction4 | 14218195 | 12417460 | 0.873349958 |
| P5-fraction5 | 37835882 | 34349085 | 0.907844173 |
| P5-fraction6 | 46490587 | 42129390 | 0.906191828 |
| P6-fraction1 | 30520368 | 27372662 | 0.896865398 |
| P6-fraction2 | 14362188 | 12849894 | 0.894703091 |
| P6-fraction3 | 13515489 | 12077946 | 0.893637367 |
| P6-fraction4 | 14357159 | 12901071 | 0.898581049 |
| P6-fraction5 | 43268282 | 39098014 | 0.90361836 |
| P6-fraction6 | 45457085 | 41251379 | 0.907479637 |
| P7-fraction1 | 23067904 | 20908884 | 0.906405888 |
| P7-fraction2 | 12925106 | 11618873 | 0.898938314 |
| P7-fraction3 | 14209199 | 12767388 | 0.898529748 |
| P7-fraction4 | 11530395 | 9782437 | 0.848404326 |
| P7-fraction5 | 33948750 | 30821543 | 0.907884473 |
| P7-fraction6 | 19070763 | 15298701 | 0.802207075 |
| P8-fraction1 | 21067261 | 18462024 | 0.876337175 |
| P8-fraction2 | 12076381 | 10331913 | 0.855547121 |
| P8-fraction3 | 14692230 | 12613209 | 0.858495205 |
| P8-fraction4 | 12486538 | 11002695 | 0.881164579 |
| P8-fraction5 | 22157769 | 19919925 | 0.899004092 |
| P8-fraction6 | 21613890 | 19613511 | 0.907449376 |
| P9-fraction1 | 28620850 | 26004732 | 0.90859398 |
| P9-fraction2 | 13343983 | 12044120 | 0.90258808 |
| P9-fraction3 | 16122378 | 13777074 | 0.854531137 |
| P9-fraction4 | 11183883 | 9421393 | 0.842408044 |
| P9-fraction5 | 38413663 | 34961766 | 0.910138822 |
| P9-fraction6 | 30390803 | 27547396 | 0.90643857 |

These fractions were approximately 20 million (range 9.4-42.1 million) mappable reads, and 88.0% (range 71.9%-91.0%) mappable ratio. Mappable ratio= Mappable reads/Total reads.

Table S3. ctDNA content in six fractions and whole plasma from 9 SCLC patients

| Patient# | | Fraction 1 | | | Fraction 2 | | Fraction 3 | | Fraction 4 | | Fraction 5 | | Fraction 6 | | Whole plasma | |
| --- | --- | --- | --- | --- | --- | --- | --- | --- | --- | --- | --- | --- | --- | --- | --- | --- |
| 1  2 | | 1.7 | | 3.5 | | | 6.5 | | 4.9 | | 7.2 | | 10.8 | | 6.7 | |
|  |  | 8.4 | | 6.0 | | | 9.4 | | 10.1 | | 9.6 | | 17.0 | | 12.7 | |
| 3  4 | | 9.3 | | 16.9 | | | 17.3 | | 16.0 | | 18.0 | | 18.1 | | 17.4 | |
|  |  | 23.1 | | 22.8 | | | 23.5 | | 24.1 | | 23.7 | | 25.5 | | 25.8 | |
| 5  6 | | 11.1 | | 17.4 | | | 21.3 | | 20.6 | | 20.1 | | 27.0 | | 23.0 | |
|  |  | 25.8 | | 21.6 | | | 20.2 | | 23.5 | | 26.9 | | 32.4 | | 28.7 | |
| 7  8 | | 0.4 | | 13.0 | | | 10.6 | | 16.8 | | 20.5 | | 37.2 | | 23.3 | |
|  |  | 27.1 | | 21.8 | | | 30.5 | | 18.4 | | 32.4 | | 36.9 | | 35.7 | |
| 9 | | 2.3 | | 5.2 | | | 37.9 | | 38.6 | | 40.5 | | 40.1 | | 41.2 | |
|  | |  | | |  | |  | |  | |  | |  | |  |  |

Table S4. Selected segments and absolute Log2 ratio from fraction 6 and whole plasma

| Patient # | Samples | Selected segments | Log2 ratio (absolute) | ctDNA content (%) |
| --- | --- | --- | --- | --- |
| 1 | Fraction 6 | 6q | 0.16 | 10.8 |
|  | Whole plasma | 6q | 0.1 | 6.7 |
| 2 | Fraction 6 | 16q | 0.27 | 17 |
|  | Whole plasma | 16q | 0.2 | 12.7 |
| 3 | Fraction 6 | 22q | 0.29 | 18.1 |
|  | Whole plasma | 22q | 0.28 | 17.4 |
| 4 | Fraction 6 | 15q | 0.42 | 25.5 |
|  | Whole plasma | 15q | 0.43 | 25.8 |
| 5 | Fraction 6 | 3p | 0.45 | 27 |
|  | Whole plasma | 3p | 0.38 | 23 |
| 6 | Fraction 6 | 13q | 0.56 | 32.4 |
|  | Whole plasma | 13q | 0.49 | 28.7 |
| 7 | Fraction 6 | 16p | 0.67 | 37.2 |
|  | Whole plasma | 16p | 0.38 | 23.3 |
| 8 | Fraction 6 | 1p | 0.66 | 36.9 |
|  | Whole plasma | 1p | 0.64 | 35.7 |
| 9 | Fraction 6 | 13q | 0.74 | 40.1 |
|  | Whole plasma | 13q | 0.77 | 41.2 |

Table S5: Clinical characteristics of NSCLC patients.

| **Characteristic** | **N = 22 (%)** |
| --- | --- |
| **Age, yr** |  |
| < 65 | 12 (54.5%) |
| ≥ 65 | 10 (45.5%) |
| **Gender** |  |
| Male | 16 (72.7%) |
| Female | 6 (27.3%) |
| **TNM Stage** |  |
| I/II | 3 (13.6%) |
| III/IV | 19 (86.4%) |
| **CEA** |  |
| Positive | 8 (36.4%) |
| Negative | 14 (63.6%) |
| **CYFRA 21-1** |  |
| Positive | 9 (40.9%) |
| Negative | 13 (59.1%) |
| **Tissue based EGFR genotyping** |  |
| Exon 19 deletion | 13 (59.1%) |
| L858R | 9 (40.9%) |
